# Analysis of hard tissue facial symmetry after unilateral mandibular reconstruction

**DOI:** 10.1186/s40902-021-00299-2

**Published:** 2021-06-01

**Authors:** Mohamad Saleh Khaghaninejad, Leila Khojastepour, Hanie Ahmadi, Saeid Tavanafar, Alireza Ebrahimi, Mohammad Mahjoori-Ghasrodashti

**Affiliations:** 1 Department of Oral and Maxillofacial Surgery, School of Dentistry, Shiraz University of Medical Sciences, Ghasrodasht St, Shiraz, Iran; 2 Department of Oral and Maxillofacial Radiology, School of Dentistry, Shiraz University of Medical Sciences, Shiraz, Iran; 3 Student Research Committee, School of Dentistry, Shiraz University of Medical Sciences, Shiraz, Iran; 4 Eastman Institute for Oral Health, Rochester, USA

**Keywords:** Mandibular reconstruction, Gonion point, Facial asymmetry, Hard tissue

## Abstract

**Background:**

This study aimed to determine how successful reconstruction of the mandible can recover the symmetry.

**Materials and methods:**

All patients who underwent surgical treatment for unilateral mandibular reconstruction in 4 years were retrospectively examined. Bilateral differences of gonion (GO) positions were measured in 3 dimensions based on immediate postoperative computed tomography. The data collected was analyzed in 3 ways: First, the comparison of bilateral differences of GO in 3 dimensions. Second, the mean Asymmetry Index in control subjects was used to divide all cases into three groups: “Symmetry,” “Asymmetry,” and “Marked asymmetry.” Third, “maximum normal asymmetry” was calculated, and all cases were categorized as below and above maximum normal asymmetry. The difference between two gonial angles was used to determine the amount of asymmetry.

**Results:**

Forty-seven patients and 47 normal adults were enrolled. The mean bilateral GO difference in the control group was higher than in the study group patients, but it was not statistically significant. The mean Asymmetry Index for the control group was not also significantly higher than the study cases. The study group was “Symmetric” in 78.7% of the cases whereas the control group in 91.4%, 19.1% of the study group and 8.5% of controls were “Asymmetric,” and 2.1% of study cases and 0% of controls were “Markedly Asymmetric.” Maximum normal asymmetry was 82.9% in the study group and 97.8% in the control group. The mean differences between the right and left gonial angles were higher in the study group, but it was not significant (*P* = 0.1).

**Conclusions:**

Our study’s results showed that bilateral symmetry in mandibular reconstruction patients was satisfactory and similar to the normal individuals.

## Introduction

Facial asymmetry is a common problem that could lead to functional and esthetic complications [1]. It is noteworthy that normal individuals could have minor and indiscernible facial asymmetries that do not cause esthetic or functional problems and do not require any treatment [2]. Thereupon, categorizing the facial asymmetries as “normal” and “abnormal” has been a challenging task for clinicians [2]. On this matter, the clinically detectable facial asymmetries are categorized into functional components (e.g., laterally deflected mandible), soft tissue (e.g., masseter hypertrophy), dental (e.g., congenital missing tooth), and hard tissue (e.g., hemifacial microsomia) asymmetries [1]. The asymmetry could occur because of congenital disorders such as cleft lip or palate, neurofibromatosis, torticollis, developmental deformities, and acquired conditions such as fibrous dysplasia, unilateral condylar hyperplasia, and facial trauma [1].

Mandibular defects might occur because of inflammatory disease (e.g., osteomyelitis), benign or malignant tumors, osteoradionecrosis, and trauma, leading to functional and facial esthetic problems [3]. In these cases, mandibular reconstruction surgery is indicated to reverse the defects’ consequences and restore facial function and form [4]. The surgeons are operating different approaches and techniques to improve the mandible’s anatomical reconstruction regarding the surgery. The appliance of reconstruction plates is a common and traditional technique to ridge a defect, stabilize residual segments, and maintain occlusion and contour [4]. However, several complications are associated with applying the plates, such as infection, wound dehiscence, plate fractures, bending of the mandible, and facial asymmetry [5, 6].

The assessments of facial asymmetry include a medical interview to evaluate the patients’ complaints and potential risk factors, extra-oral examinations to inspect facial morphology, intra-oral examinations to assess malocclusion, tipping of teeth, functional deviation mandible, and radiological assessment [7]. Traditionally, clinicians have used different imaging methods such as lateral, frontal, and submentovertex cephalograms and panoramic radiographs for radiological assessment of facial asymmetry [8]. These imaging methods have had several flaws considering the asymmetry evaluation, for instance, incapability of magnification and displaying overlapping structures [9, 10]. Currently, because of the advocacies of 3-dimensional (3D) computed tomography (CT) imaging, most clinicians prefer to use 3D-CT scans for the diagnosis and planning of treatment [11, 12]. 3D-CT imaging enables the clinicians to observe craniofacial bones from different viewing angles and determine volumetric measurements of the bones, which are tremendously valuable regarding the diagnosis and treatment planning of facial asymmetries.

As mentioned above, facial asymmetry is one of the mandible reconstruction surgery complications that could lead to esthetic (e.g., acceptable appearance) and functional (e.g., speaking and swallowing) problems. However, the lack of reports on the surgical outcomes and insufficient comprehensive references regarding evaluating these outcomes are palpable [13, 14]. Moreover, previous investigations have recommended multiple complex assessment methods and have produced inconsistent results [14, 15]. In the present study, we aimed to retrospectively evaluate the facial asymmetry of the patients who had undergone mandible reconstruction surgery and received mandible plates. We also proposed a novel technique for evaluating facial asymmetry in these patients using 3D-CT scans and calculating linear measurements of craniofacial bones.

## Material and methods

### Design and population

This retrospective cross-sectional matched-control study was done on 47 patients who undergone unilateral mandibular reconstruction surgery at our teaching hospital between March 2015 to March 2020. Among 107 patients who received the surgery and a reconstruction plate for the mandible, forty-seven were included in the present study according to the inclusions criteria, medical history, and available imaging in the picture archiving and communication system. It is worth mentioning that mandibular reconstruction’s adequacy with reconstruction plate had been approved for all the patients by the surgery team using postoperative CT scans immediately after the procedure.

Written informed consent for scientific use of patients data is usually taken preoperatively from all of the participants in our teaching hospital, and their anonymity is guaranteed. The institutional research board approved the study protocol (ID: IR.SUMS.DENTAL.REC.1399.014).

The inclusion criteria for the participants of the study were as follows: patients between 11 and 69 years of age, unilateral pathological lesions of the mandible, mandibular reconstruction by reconstruction plate, available pre- and postoperative CT scans, complete medical records, and returned for a follow-up visit. In this study population, surgical team routinely adapt the reconstruction plate to portion of mandible that deemed to be resected. The Jewer’s classification (HCL classification) that reflects the reconstructive process’s complexity was used to classify mandibular defects in this study. Mandibular defects were designated “C” when defects included both canines. “L” was designated for lateral defects excluding the condyle, and H stands for lateral segment defects of any length, including the condyle (Hemi-mandibulectomy) [16]. A combination of C, L, and H in each patient also was considered.

The exclusion criteria were the presence of distinctive facial asymmetries before the surgery (approved by three radiologists), history of cleft lip or cleft palate, post-traumatic deformities, facial bone fractures, bilateral pathologic lesions of the mandible, prior maxillofacial surgeries, removal of mandibular reconstruction plate due to infection or dehiscence, implementation of other prosthetic reconstructions rather than reconstruction plates, and inadequate imaging and medical records of treatment or follow-up visits.

The control group was selected from the patients who had obtained face and head CT scans in our hospital’s radiology ward from March 2017 to March 2020. The patients included in the control group were matched to the study sample regarding age and gender. Patients with distinctive facial asymmetry, a history of facial trauma, facial bone fracture, or craniofacial hard tissue surgery were excluded from the controls.

### Study landmarks

**Fig. 1:**
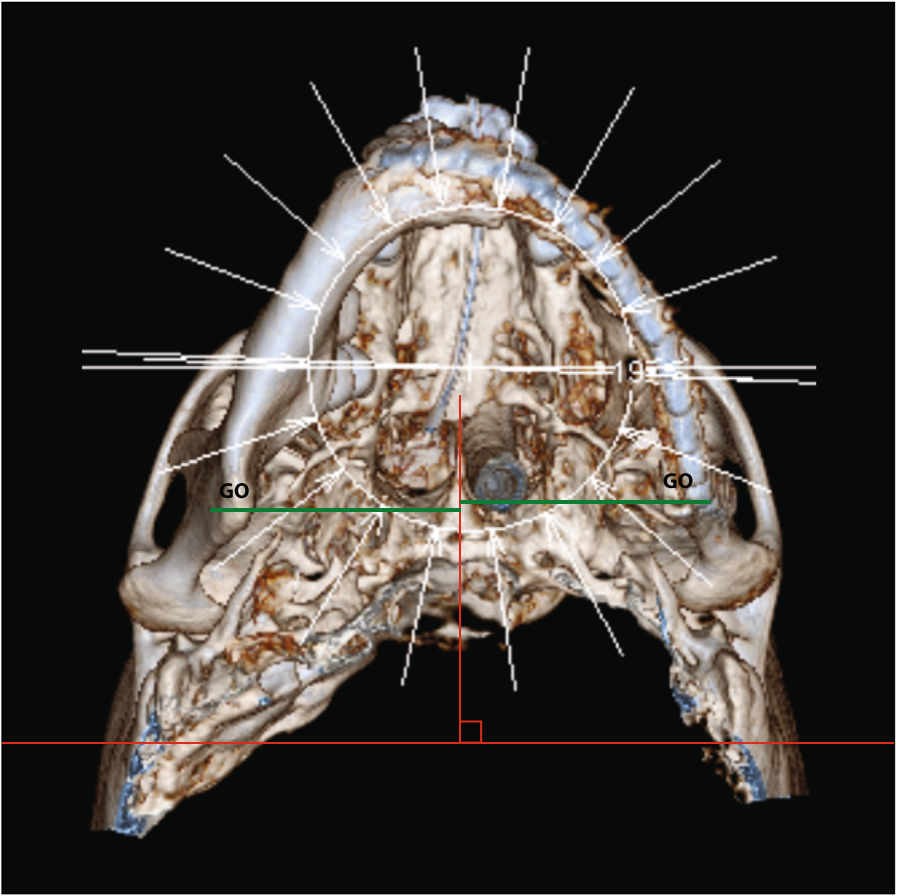
Medial-lateral distances between gonion points (GO) and vomer line (VL) in the 3D-CT image (submentovertex view) were measured and compared in all participants

**Fig. 2:**
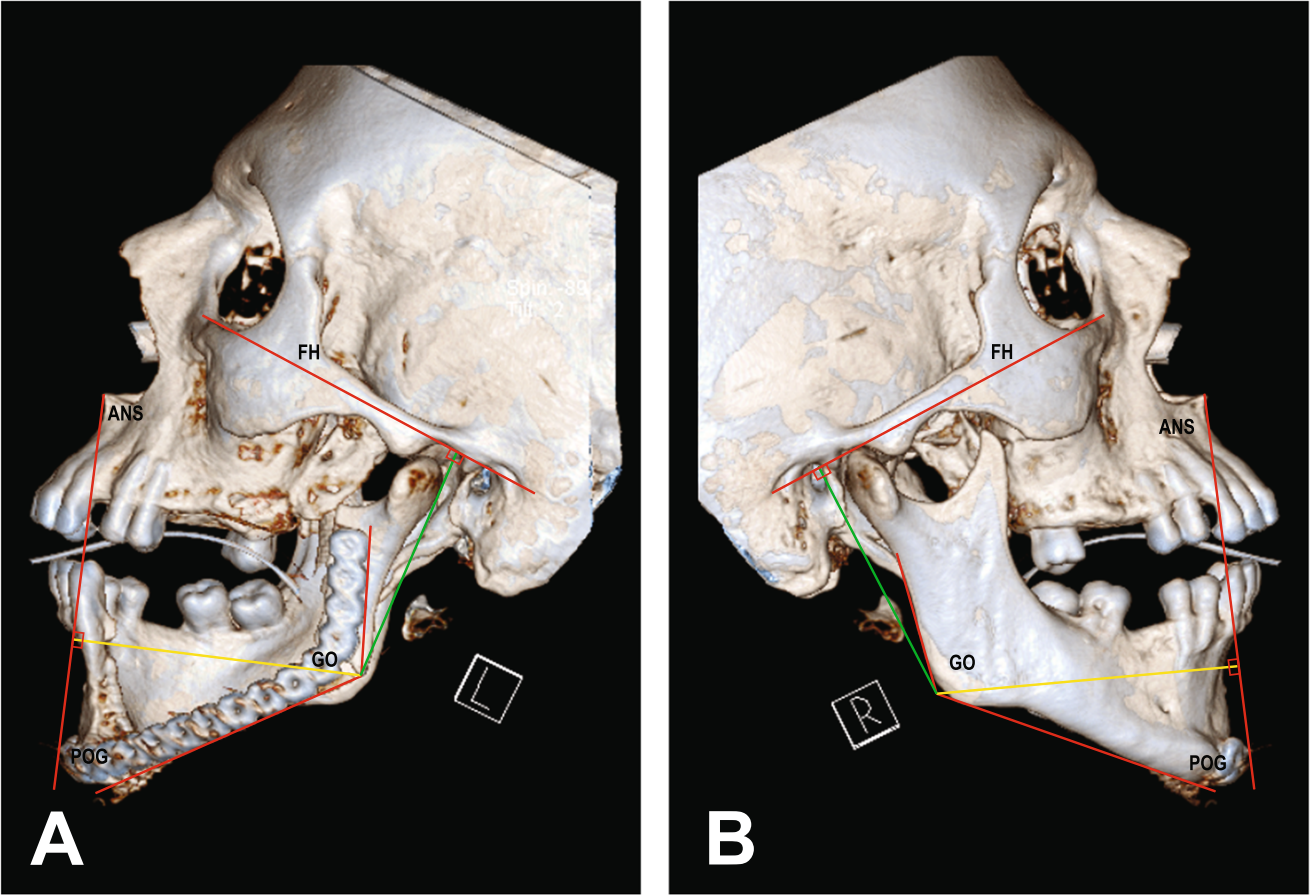
The bilateral distances of the GO point to the Frankfurt line and a line passing through the ANS and Pog were measured to assess its position in superior-inferior (green line) and anterior-posterior (yellow line) dimensions in 3D-CT (sagittal view) for **a** reconstruction cases and **b** normal control group cases

The radiological landmarks used in the present investigation to evaluate the participants’ hard tissue asymmetry are shown in Table 1. Each patient was compared with him/herself. The position of gonion (GO) in a three-dimensional (3D) CT scan was used to evaluate facial symmetry according to the previous investigations [8, 17, 18]. In this regard, the differences between GO’s position on the left and right sides were compared in three axes as the following method. The difference between the left and right GOs’ position in submentovertex 3D images was used to evaluate symmetry in the medial-lateral distance (dM-L). Differences between right and left sides in the anterior-posterior distance (dA-P) and superior-inferior distance (dS-I) were also evaluated based on lateral 3D images (sagittal view). For medial-lateral distance (dM-L), the distance between gonion and vomer line (VL) in each side was measured by drawing a perpendicular line from each gonion into (VL) (Fig. 1). Regarding the superior-inferior distance (dS-I), a perpendicular line from each GO point was drawn to the Frankfurt line, and the distances between the two sides were measured. A perpendicular line was drawn from the GO points to the line that conjoins the anterior nasal spine point (ANS) and Pogonion (Pog) point in order to calculate the anterior-posterior distance (dA-P); the differences between the two sides were measured as mentioned above (Fig. 2a, b).

**Table 1:** Definition of landmarks

**Anterior nasal spine (ANS)**	A protrusion of the maxilla at the base of the nose
**Frankfurt line (FH)**	Connected the uppermost point on the external acoustic meatus's margin with the lowermost point on the orbital margin.
**Gonion (GO)**	The point between the inferior point on the posterior border of ramus and the most posterior point on the inferior border of the mandibular body.
**Gonial angle**	The angle between the junction of the posterior border of the ramus and lower borders of the mandibular body
**Pogonion (Pog)**	The most forward-projecting point of the symphysis
**Vomer line (VL)**	The perpendicular line from the vomer to the horizontal plane (VL)

### Analysis of asymmetry

After calculating the dM-L, dA-P, and dS-I in left and right GO points, the Asymmetry Index (AI) was measured for each participant using a previously approved formula [19]. The AI was calculated for both study and control groups.

$$ \mathrm{Asymmetry}\ \mathrm{Index}=\surd {\left[\left(\mathrm{R}\right)\ \mathrm{dM}-\mathrm{L}-\left(\mathrm{L}\right)\ \mathrm{dM}-\mathrm{L}\right]}^2+{\left[\left(\mathrm{R}\right)\ \mathrm{dA}-\mathrm{P}-\left(\mathrm{L}\right)\ \mathrm{dA}-\mathrm{P}\right]}^2+{\left[\left(\mathrm{R}\right)\ \mathrm{dS}-\mathrm{I}-\left(\mathrm{L}\right)\ \mathrm{dS}-\mathrm{I}\right]}^2 $$

L = Left gonion point, R = Right gonion point

The mean and standard deviation (SD) of AIs in the control subjects were used to calculate a baseline value for further evaluations. The participants were classified into three groups as “symmetry,” “asymmetry,” and “marked asymmetry,” according to the baseline value. The participants were labeled as “symmetry” if the AI was below the baseline value. They were labeled as “asymmetry” when the AIs were between the baseline and twice the baseline value and as “marked asymmetry” when the AIs were twice the baseline value [8]. Moreover, we compared the participants using “maximum normal asymmetry,” which categorizes the participants as below and above maximum normal asymmetry according to the asymmetry threshold (maximum normal asymmetry = mean _control_ + 2SD _control_) [20].

**Fig. 3:**
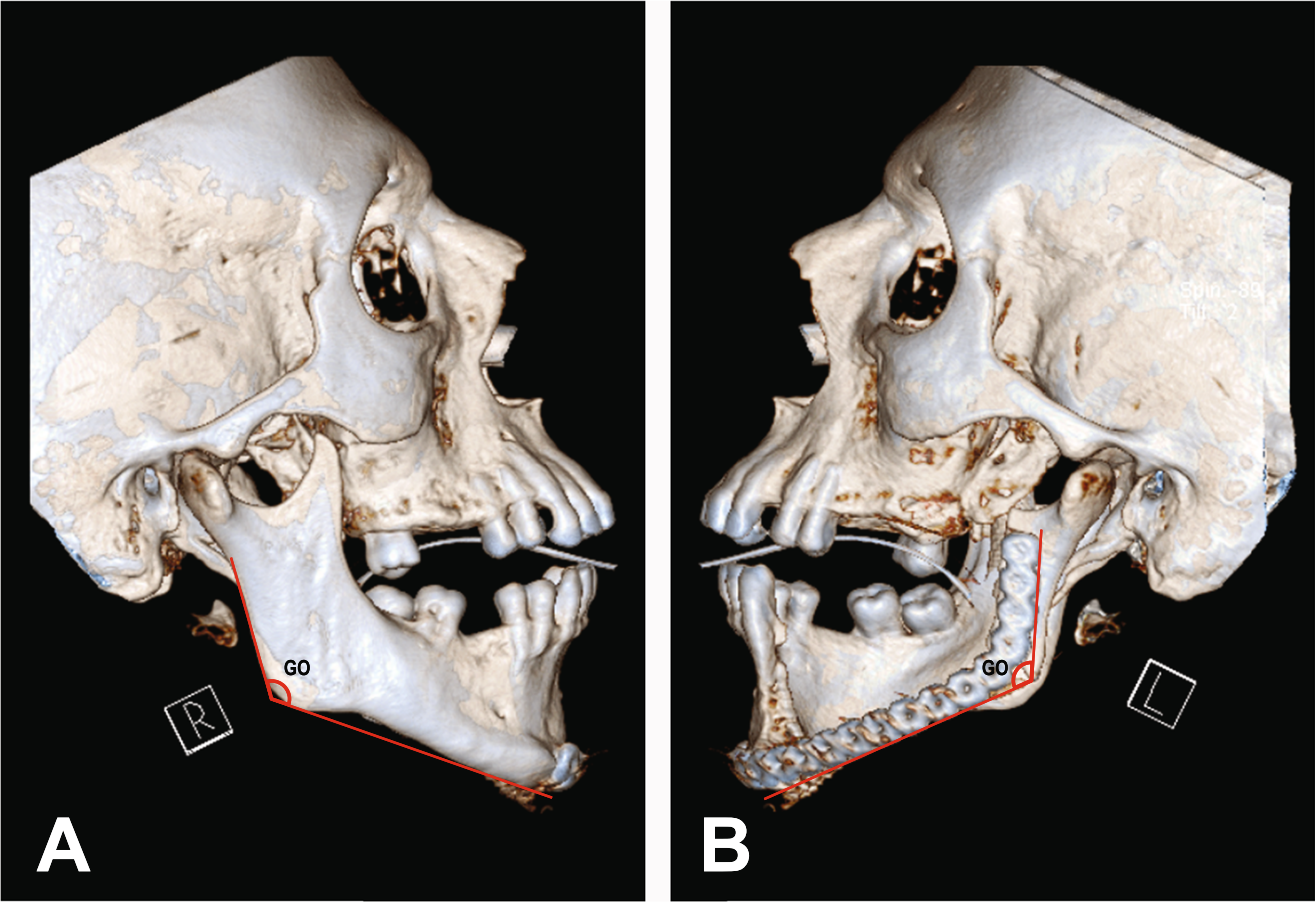
**a** The measurement of a non-affected gonial angle in 3D-CT images (sagittal view), and **b** for reconstructed gonial angle

We also used angular measurements to evaluate the degrees of asymmetry in the participants. The gonial angle differences between the right and left mandibles were measured and used to determine the angular asymmetry as the following [18] (Fig. 3a, b). Angular asymmetries could be non-significant (NS) (>0° and <3°), light (L) (≥3 and <5), moderate (M) (≥5 and <10), or severe (S) (≥ 10).

### Data gathering and analysis

All measurements were done using picture archiving and communication system (PACS) filmless radiology software (Version. 4, INFINITT, North America, Inc) with 0.1-mm accuracy. Data were analyzed using the Statistical Package for Social Sciences (SPSS, version. 26.0, SPSS Inc., Chicago, IL, USA).

The same investigator carried out the measurements twice, with at least a 48-h inter-session period. The results of the first and second measurements were compared using intra-class correlation. Mean ± SD was calculated for the inferential statistics, and the data were compared using the two-tailed T-test. A *p*-value of < 0.05 was considered statistically significant, with a 95% reliability.

## Result

There was no statistically significant difference between the first and second measurements. Correlations between the first and second measurements ranged from 0.856 to 0.928. Forty-seven patients who had undergone unilateral mandibular reconstruction surgery and received a reconstruction plate, including 16 females and 31 males with a mean age of 41.74±14.8 years (age range 13–67 years old), enrolled in the present research. The control group was comprised of 47 individuals with age and gender-matched subjects.

Twenty-five of 47 patients had received a left and 22 had received a right-sided mandibular reconstruction plate. According to Jewer’s classification, 11 (23.4%) of patients were categorized in the H group, 27 (57.4%) in the L group, 1 (2.2%) in the HC group, and 8 (17%) in the LC group.

Table 2 shows the mean differences in the left and right GO points in each dimension. In the study group, the dM-L, dS-I, and dA-P were calculated as 3.68 mm, 4.22 mm, and 5.38 mm, respectively, while these measures were 4.37mm, 5.06mm, and 6.35mm for the control group. Our results indicated that the patients who had done the mandibular reconstruction surgery had lower asymmetry than the control subjects; however, it was not significant (*p* > 0.05).

**Table 2:** Differences between the position of right and left gonions in different dimensions

Measurement (mm)	Study group		Control group		*P*-value
	Mean (range)	SD	Mean (range)	SD	
M-L	3.68 (0–17)	3.35	4.37 (0–9.4)	2.02	0.22
S-I	4.22 (0–20)	4.09	5.06 (0–9)	2.50	0.23
A-P	5.38 (0–17)	4.86	6.35 (0–12)	3.02	0.24

M-L, S-I, and A-P represent mediolateral, superior-inferior, and anterior-posterior dimensions respectively; SD, standard deviation (a *p*-value of < 0.05 was considered statistically significant)

The mean of AIs was 9.81 ± 2.87 for the control group and 8.90 ± 5.6 for the study group, which shows that normal controls have higher asymmetries than the patients; however, it was not significant (*p*-value = 0.33). The patient’s classification’s standard baseline value was calculated to be 12.68 mm in the present investigation. According to this baseline value, nearly four-fifths of the patients had symmetrical mandible, 9 (19.1%) had mandibular asymmetry, and 1 (2.1%) had a marked asymmetry (Table 3).

**Table 3:** Comparison between study and control groups based on AI (Asymmetry Index) and gonial angle

		Study group ***N*** = 47	Control group ***N*** = 47
**Symmetry based on AI**	Symmetry	37 (78.7%)	43 (91.4%)
	Asymmetry	9 (19.1%)	4 (8.5%)
	Marked asymmetry	1 (2.1%)	0 (0%)
**Gonial angle asymmetry**	Not significant	7 (14.9%)	8 (17%)
	Light	16 (34%)	21 (44.7%)
	Moderate	20 (42.6%)	16 (34%)
	Severe	4 (8.5%)	2 (4.3%)

In the present investigation, the maximum standard asymmetry value was calculated as 15.55 mm. According to this value, more than four-fifths of the patients (82.9%) and 46 (97.8%) of the control group had symmetrical mandibles, though 8 (18%) of the study group and 1 (2%) of the standard controls were labeled as having asymmetrical mandibles.

The mean differences between the right and left gonial angles are 2.25 ± 0.79 for normal controls and 2.44 ± 0.85 for the patients. The minimum and maximum of the differences were measured 0° and 15° for the study group, and 1° and 11° for the control group. Considering the angular symmetry, the standard controls had higher symmetry rates; however, it was not significant (*p* = 0.1).

The severity of the gonial angle’s angular asymmetry was also determined for the participants (Table 3). In the study group, 4 (8.5%), 20 (42.6%), 16 (34%), and 7 (14.9%) of the patients had severe, moderate, light, and non-significant angular asymmetries, respectively. These numbers were 2 (4.3%), 16 (34%), 21 (44.7%), and 8 (17%) for the normal controls, respectively. As these results show, the number of patients with severe angular asymmetry was higher in patients who underwent mandibular reconstruction surgery.

## Discussion

GO has been used as a critical point for the evaluation of facial asymmetry after mandibular reconstruction surgeries [17]. Moreover, 3D-CT imaging is preferred for assessing hard-tissue landmarks as it could 3-dimensionally evaluate the landmarks [19]. In this regard, cone beam computed tomography (CBCT) is also recommended since it has higher image quality and lower radiation [21–23]. Recently, 3D stereophotogrammetry has been proposed to evaluate facial symmetry evaluation after mandibular reconstruction [3]. In the present investigation, we have introduced a novel method for evaluating facial symmetry after mandibular reconstruction. We used head and face 3D-CT scans for linear and angular right and left GO points and calculated the differences between the two points.

The mean of differences between right and left GO in M-L, S-I, and A-P dimensions were 3.68 mm, 4.22 mm, and 5.38 mm, respectively, in the study group. While these numbers were 4.37 mm, 5.06 mm, and 6.35 mm, respectively, in the control group. Our result shows that the patients who underwent mandibular reconstruction surgery had lower facial asymmetry than normal individuals; however, the rates were not statistically significant. These lower rates of facial asymmetry in the study group could be because of the high prevalence of facial asymmetry in the individuals that surgical interventions could correct. It is noted that the normal adult individuals might have a range of 0–12-mm differences between the right and left skeletal landmarks, using posteroanterior cephalograms [24].

Our study showed that the normal adult participants in the control group had hard tissue facial asymmetry, which was evaluated by linear measurement. It is consistent with previous studies which have mentioned that facial asymmetry is a prevalent problem even in normal individuals [25, 26]. In this regard, an investigation showed that moderate to severe mandibular asymmetry could be found in normal children [18]. Another study demonstrated that mild facial hard tissue and right-sided asymmetry could be frequently found in the average population [27]. On the other hand, the lower third of the face and the lateral landmarks are commonly involved in facial asymmetries; for example, the chin, ramus, and GO point show higher asymmetry [25, 26], and it has been shown that 74% of 1460 patients who were referred for orthodontic surgery had lower third facial asymmetry [28]. The average population might have facial asymmetries that do not cause any functional or esthetic complications, categorized as “normal” asymmetry.

In the present study, the patients who underwent mandibular reconstruction surgery with a reconstruction plate had lower AI rates than the control group participants. Moreover, our study showed that condylar involvement does not affect the outcome of facial symmetry. The mean of AI was calculated 9.81±2.87 in the normal controls group and 8.90 ± 5.6 in the study group patients. This result is consistent with prior reports indicating high AI scores could be found in normal individuals, especially in the lower face [8, 29]. Previous reports also showed that lower third facial symmetry had significantly improved in the patients with class II deformity after bimaxillary surgery [30]. The 1-year follow-up of the patients with Class III deformity who had undergone combined Le Fort I and a bilateral sagittal split osteotomy showed the same results [31]. As the patients’ facial AIs have lowered, their satisfaction has improved after the surgery. Careful pre-surgery analysis of the underlying defects is needed to predict the restored mandible’s accurate size and continuity and design the correct plate [32, 33]. It seems that the maxillofacial surgeries—if indicated—could generally improve facial asymmetries.

According to the AI scores, we categorized the participants into three groups of “Symmetry,” “Asymmetry,” and “Marked Asymmetry,” based on a previously practiced method [8]. Considering this classification, nearly 20% of the study group patients and 10% of the normal adult participants in the control group had facial asymmetry. One of the patient (2.1%) showed marked asymmetry, while no average individual showed marked facial asymmetry. It is noteworthy that the categorization method is based on the mean of AI scores in the normal control group; hence, the prior result might not be controversial to the above results. However, according to this result, we could also suggest that facial asymmetry could be higher in patients who underwent mandibular reconstruction surgery than normal control group. We also showed that about 20% of the patients in the study group and 2% of the individuals in the control group had facial asymmetry, according to the “maximum normal asymmetry” parameter [20]. We believe these categorization methods might be more definite regarding the patients’ evaluation, as generally, we expect more facial asymmetry in patients who had done a reconstruction surgery [34].

Our investigation consistently showed that more than half of the patients in the study group who underwent surgery had moderate or severe gonial angle asymmetries. The prevalence of angular asymmetry in the normal population seems lower; however, no significant difference was found between them. Previous studies have also reported that the prevalence of gonial angle asymmetry is relatively high because of several causes, such as unilateral chewing habits and muscular atrophies [19, 25, 35].

Our study results were consistent with the previous investigations and revealed that the normal population might have mild degrees of facial asymmetries. The mean scores of AIs in the normal control group were calculated to be 9.81 ± 2.87 in the present investigation. Moreover, the angular asymmetry (light, moderate, and severe) prevalence was relatively high (83%) in these participants. On the other hand, we revealed that nearly 20% of the patients who underwent mandibular reconstruction surgery with reconstruction plate might show facial asymmetry, and around 2% of them might suffer from a marked facial asymmetry. However, we did not find any significant difference between the prevalence of facial asymmetry among the patients in the study group and the control group, indicating that the procedure has a few adverse effects in this regard.

The results of our study may have been affected by limitations. One of our study’s most significant weaknesses is that our measurements considered underlying hard bony tissue and reconstruction plate adaptation in lateral and SMV view for comparison. Although asymmetries in the underlying structure may affect overlying soft tissue, further studies required to analyze their relationship and how differences in soft tissue in different individuals would respond to underlying structures. Another limitation could be our control group which might not be the representation of whole population because no CT scan images are taken without a specific diagnostic reason. We reviewed 107 CT images to find subjects with typical head and face morphology. Furthermore, all cases in this study were referred to the teaching hospital, and the sample might not be representation of the whole population. Another restriction is that all of our participants were Iranian, while different ethnicities should be investigated to estimate the range of asymmetry in normal individuals. The use of 3D-CBCT instead of 3D-CT images should have given us more details. A rise in the number of participants of both genders and different age range is recommended to evaluate any association between age and sex and facial asymmetry.

## Conclusion

Craniofacial asymmetry is a common diagnosis even in the normal population, and in most cases, it does not have any serious consequence. The present investigation demonstrated that the mean facial asymmetry seems lower in the patients who underwent mandibular reconstruction than the normal population. This might be because of the high prevalence of asymmetry in normal individuals. However, about 21% of the study group had facial asymmetry, and the differences between right and left gonial angles were higher in the study group. Overall, it seems the outcome of the mandibular reconstruction surgery is acceptable considering the patients’ facial symmetry. More studies should evaluate facial asymmetry prevalence in normal individuals and the patients who received maxillofacial surgeries.

## Data Availability

Corresponding author is responsible for availability of data upon reasonable requests.

## Abbreviations

CT: Computed tomography
3D-CBCT: Three-dimensional cone beam computed tomography
3D-CT: Three-dimensional computed tomography
GO: Gonion
dA-P: Anterior-posterior distance
dS-I: Superior-inferior distance
dM-L: Medial-lateral distance
VL: Vomer line
ANS: Nasal spine point
Pog: Pogonion
AI: Asymmetry Index
SD: Standard deviation
